# Hemicellulosic biomass conversion by Moroccan hot spring *Bacillus paralicheniformis* CCMM B940 evidenced by glycoside hydrolase activities and whole genome sequencing

**DOI:** 10.1007/s13205-021-02919-0

**Published:** 2021-07-22

**Authors:** Soufiane Maski, Serigne Inssa Ngom, Bahia Rached, Taha Chouati, Mohamed Benabdelkhalek, Elmostafa El Fahime, Mohamed Amar, Christel Béra-Maillet

**Affiliations:** 1 Laboratoire de Microbiologie et Biologie Moléculaire, Centre National pour la Recherche Scientifique et Technique, Rabat, Morocco; 2 Université Paris-Saclay, INRAE, AgroParisTech, Micalis Institute, Jouy-en-Josas, France; 3 Collections Coordonnées Marocaines de Microorganismes, Centre National pour la Recherche Scientifique et Technique, Rabat, Morocco; 4 Département de Biologie, Faculté des Sciences, Université Mohammed V, Rabat, Morocco; 5 Plateforme de biologie moléculaire et Génomique Fonctionnelle, Centre National pour la Recherche Scientifique et Technique, Rabat, Morocco

**Keywords:** *Bacillus paralicheniformis*, Whole genome, Hemicellulose, Glycoside hydrolase, Lichenan, Xylan

## Abstract

Thermophilic bacteria, especially from the genus *Bacillus*, constitute a huge potential source of novel enzymes that could be relevant for biotechnological applications. In this work, we described the cellulose and hemicellulose-related enzymatic activities of the hot spring *Bacillus aerius* CCMM B940 from the Moroccan Coordinated Collections of Microorganisms (CCMM), and revealed its potential for hemicellulosic biomass utilization. Indeed, B940 was able to degrade complex polysaccharides such as xylan and lichenan and exhibited activity towards carboxymethylcellulose. The strain was also able to grow on agriculture waste such as orange and apple peels as the sole carbon source. Whole-genome sequencing allowed the reclassification of CCMM B940 previously known as *B. aerius* into *Bacillus paralicheniformis* since the former species name has been rejected. The draft genome reported here is composed of 38 contigs resulting in a genome of 4,315,004 bp and an average G + C content of 45.87%, and is an important resource for illuminating the molecular mechanisms of carbohydrate metabolism. The annotated genomic sequences evidenced more than 52 genes encoding glycoside hydrolases and pectate lyases belonging to 27 different families of CAZymes that are involved in the degradation of plant cell wall carbohydrates. Genomic predictions in addition to in vitro experiments have revealed broad hydrolytic capabilities of the strain, thus reinforcing its relevance for biotechnology applications.

**Supplementary Information:**

The online version contains supplementary material available at 10.1007/s13205-021-02919-0.

## Genome research reports

Lignocellulosic biomass, mainly composed of cellulose, hemicelluloses and lignin, is the most abundant complex biopolymer in the nature. It is of major interest for biotechnology as this feedstock is used for the production of renewable biofuels and other high-value chemicals, through an enzymatic saccharification process (Zhang et al. 2018). However, depolymerization of lignocellulose is challenging and it requires high-performance enzymes that act in synergy (Houfani et al. 2020). Therefore, enzymes produced by microorganisms, such as bacteria, are used for the deconstruction of the complex polysaccharides of cellulose and hemicelluloses which are tightly bound together in plant cell walls (Zeng et al. 2017).

Xylan is the main component of hemicelluloses and consists of a β-1,4-d-xylan backbone with short side chains of *O*-acetyl, β-l-arabinofuranosyl, d-α-glucuronic acid and phenolic acid (Biely et al. 2016). Other polysaccharides such as xyloglucan, lichenan and mannans are part of the hemicellulolytic fraction of plant cell walls. Xylanases, xyloglucanases, lichenases, and endoglucanases are necessary to completely degrade these polysaccharides into simple sugars to allow glycolytic fermentation to produce energy. These enzymes belong to one of the glycoside hydrolase (GH) families, a subgroup of the carbohydrate-active enzymes (CAZymes) (Lombard et al. 2014) and very often associated with carbohydrate-binding modules (CBM) that facilitate adhesion of enzymes to cellulose or hemicelluloses.

Most microorganisms living in extreme environmental habitats such as hot springs have been considered attractive producers of lignocellulolytic enzymes for industrial bioconversion processes (Thapa et al. 2020). Morocco has more than 20 hot springs distributed in different regions (Cidu and Bahaj 2000; Bouchaou et al. 2017) which are suitable habitats for thermophilic microorganisms growing optimally between 55 and 80 °C. In addition, thermophilic bacteria are known for their ability to produce thermostable enzymes of biotechnological interest (Knapik et al. 2019). Previously, Aanniz et al. (2015) investigated the diversity of thermophilic bacteria in 4 different hot springs in Morocco, which enabled the isolation of 79 bacterial strains belonging to the genus *Bacillus* (www.ccmm.ma). Some of these strains were identified as *Bacillus aerius*. They grow optimally at 55 °C and exhibit amylolytic, proteolytic or cellulolytic activity (Aanniz et al. 2015). However, strains belonging to *B. aerius* species should be reclassified as this species name has been rejected since 2015 (Dunlap 2015). For this reason, we chose the CCMM B940 strain as the representative of the *B. aerius* group isolated by Aanniz et al. (2015) from a hot spring for biological characterization of its potential hemicellulolytic and cellulolytic activities and whole-genome sequencing analysis.

**Fig. 1:**
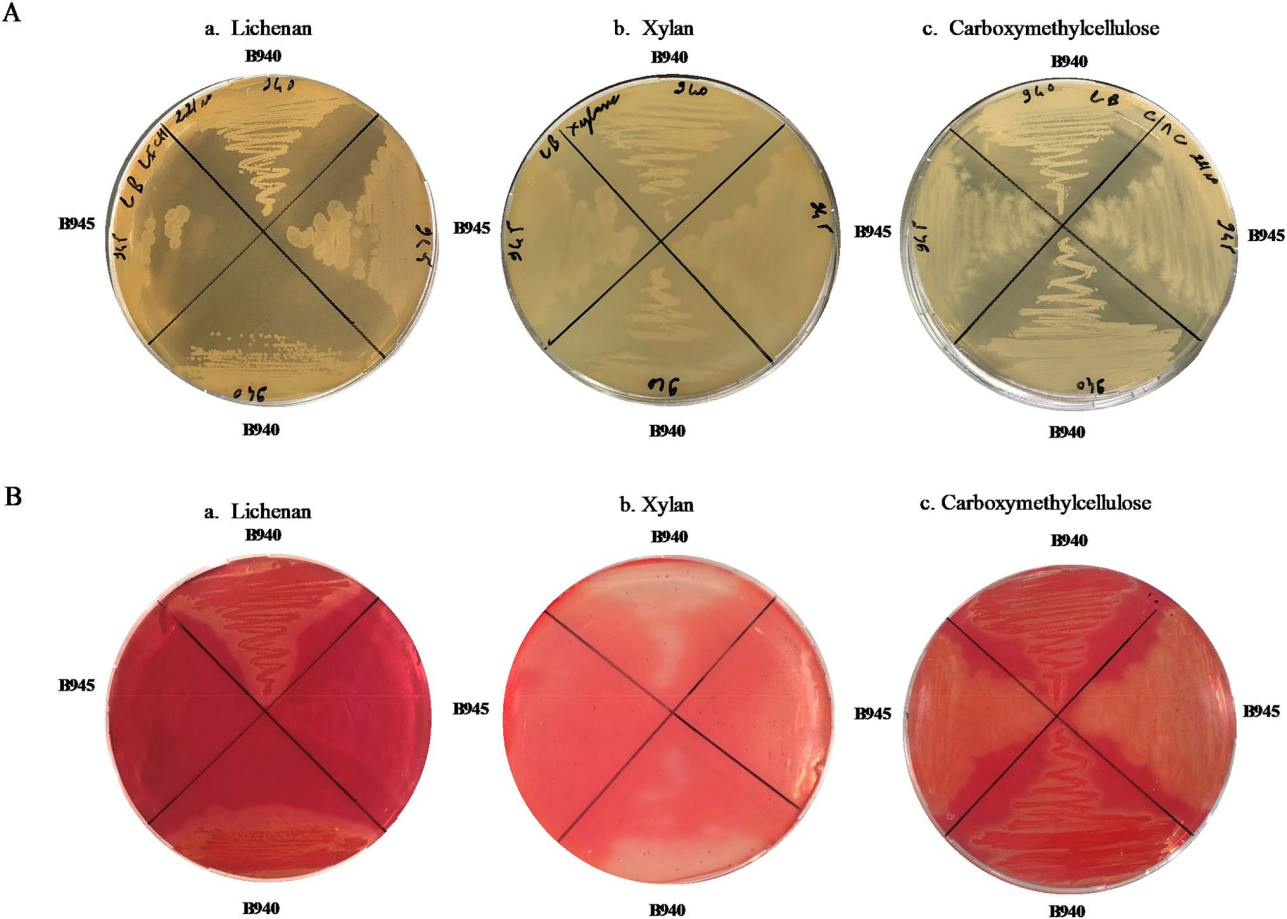
Growth **A** and detection of lichenase (a), xylanase (b) and carboxymethylcellulase (c) activities **B** of *Bacillus* CCMM B940 and CCMM B945 strains cultivated in LB agar streak plates supplemented with 0.5% (w/v) lichenan, xylan or carboxymethylcellulose (CMC), respectively. Plates were incubated 18 h at 37 °C. Congo red staining was performed as described in Patrascu et al. (2017). Clear halos around the streaks indicate the degradation of the corresponding polysaccharide.

B940 strain was grown twice on Luria–Bertani (LB) agar medium (BD Difco, Sparks, Maryland, USA) for 18 h at 37 °C under aerobic conditions. Individual colonies were then picked from LB plates and cultivated at 37 °C for 18 h in LB agar medium supplemented with 0.5% Icelandic moss Lichenan (Megazyme, Wicklow, Irlande), beechwood Xylan (Megazyme, Wicklow, Irlande), or medium viscosity Carboxymethylcellulose (CMC, Sigma, MO, USA), respectively. A second bacterial strain (CCMM B945) from the same *B. aerius* group, known for inability to degrade lichenan and xylan (data not shown) was used as a negative control for degradation of hemicellulose and following the same experimental protocol as for CCMM B940. Both strains grew well on LB agar medium supplemented with purified fibres (Fig. 1A).

To reveal whether the strains were able to degrade the complex polysaccharides added into the medium, the plates were stained with 0.1% Congo red as previously described (Patrascu et al. 2017). Clear halos around the streaks indicated that B940 strain can degrade all three polysaccharides, and thus capable of producing the appropriate enzymes. As expected, lichenases and xylanases were only produced by the CCMM B940 strain (Fig. 1B). Strain CCMM B945 exhibited significant activity against CMC, indicating production of endoglucanases, similar to the CCMM B940 strain.

The Congo red test demonstrated that live cells of the B940 strain exhibited lichenase and xylanase activities and also produced endoglucanases for glucan-polymer degradation. However, to evaluate biomass utilization potential of this strain for biotechnology applications, we searched for the presence of those enzymes in the rather easily accessible B940 strain supernatants. For this purpose, the strain was cultivated in the basal salt solution (BSS), a chemically defined medium modified from Parab et al. (2017), with a lower salt concentration (NaCl 6 g L^−1^). The medium was supplemented with fruits peels (orange or apple) as the sole source of carbon and then sterilized 15 min at 121 °C. Fruit peels are a rich source of cellulose, hemicellulose, lignin and pectin (Rivas et al. 2008; Joglekar et al. 2019). Orange peel is rich in structural polysaccharides, including cellulose (18–20%), hemicellulose (14–16%), pectin (20–22%) and lignin (5–7%) (de la Torre et al. 2019). Apple residues typically contain 7.2–43.6% cellulose, 4.3–24.4% hemicellulose, 15.2–23.5% lignin and 3.5–14.32% pectin (Dhillon et al. 2013). Both substrates were chosen because of their abundance as industrial waste around the world (de la Torre et al. 2019).

A single colony of CCMM B940 and CCMM B945 from LB agar medium was picked to inoculate 5 mL of LB Broth (Difco) and incubated overnight at 37 °C (with continuous stirring at 170 rpm), followed by second culture in 5 mL of BSS medium supplemented with 0.5% apple or orange peels and inoculated at 1%. Before adding them in the medium, apple and orange peels were dried for 24 h at 60 °C and crumbled finely using an electric grinder. After 48 h of incubation at 37 °C with stirring (170 rpm), both strains grew well in the BSS supplemented with fruit peels representing agro-industrial wastes as shown in Figure S1. Additionally, this test confirmed the ability of CCMM B940 and CCMM B945 strains to break down fibres from the peels and use the degradation products.

Protein samples from B940 and B945 strains cultivated for 48 h at 37 °C in 30 mL BSS medium supplemented with 0.5% orange and apple peels were prepared to detect and quantify hemicellulolytic and cellulolytic activities in the extracellular fluids. Briefly, cultures were centrifuged for 15 min at 5000×*g* and 4 °C, supernatants were filtered through a 0.22 μm Millipore filter, and then concentrated 25-fold in a Spin-X UF10K concentrator following the supplier’s protocol (Corning B.V., Amsterdam, The Netherlands).

**Fig. 2:**
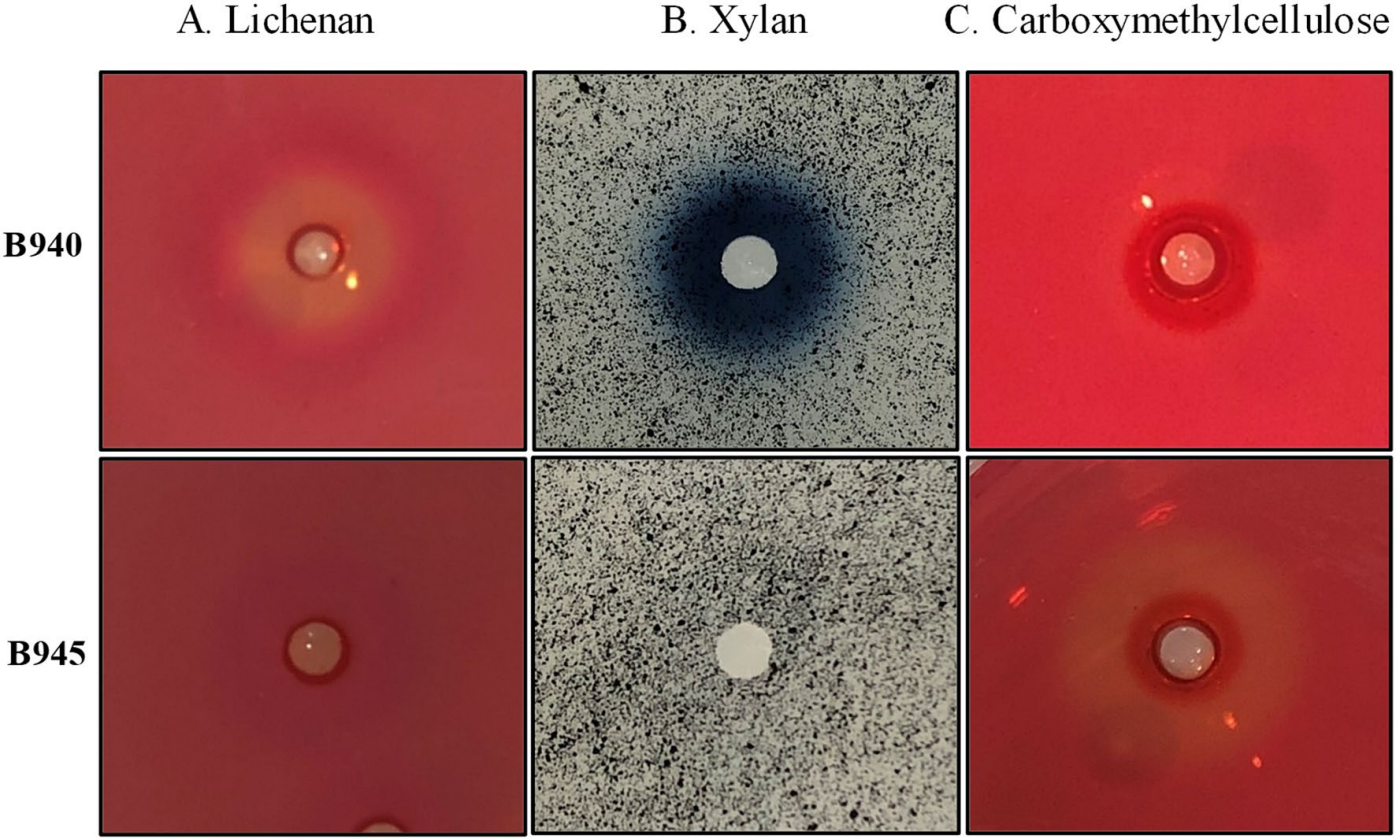
Detection of lichenase **A**, xylanase **B** and carboxymethylcellulase **C** activities of *Bacillus* CCMM B940 and B945 strains, using agar-well plate assays with concentrated extracellular proteins of BSS cultures with 0.5% apple. Agar plate BSS medium was supplemented with 0.5% (w/v) lichenan, carboxymethylcellulose (CMC), or 0.1% AZCL beechwood xylans. Plates were incubated overnight at 37 °C. Clear and blue halos around the wells indicate a positive sample for the corresponding glycoside hydrolase activity

Agar-well plate enzymatic assays were performed to detect xylanase, lichenase and endoglucanase activities using concentrated extracellular proteins. Concentrated extracellular proteins from CCMM B940 and control strain CCMM B945 were prepared in the same way. The samples were loaded (60 μL/well) in Petri dishes containing BSS medium supplemented with either 0.1% chromogenic AZCL-Xylan (Megazyme, Wicklow, Ireland), 0.5% non-chromogenic CMC (Sigma-Aldrich, MO, USA) or lichenan (Megazyme) polysaccharides. The plates were incubated overnight at 37 °C and those with non-chromogenic polysaccharide were stained with Congo red as described above. Clear and blue halos around the wells indicated that the CCMM B940 cultivated with apple peels was able to produce lichenase (Fig. 2A), endoglucanase (Fig. 2C, although weakly in the tested conditions), and xylanase (Fig. 2B). The same results were obtained with orange peels (not shown).

The CCMM B940 strain is able to grow at 55 °C in Tryptic Soy broth (Aanniz et al. 2015) and we also observed that the strain is capable of growing in BSS medium supplemented with lichenan and xylan at the same temperature (data not shown), thus reinforcing the interest of its characteristics for industrial applications requiring thermostable enzymes.

In contrast to CCMM B940 strain results, the control strain CCMM B945 was not able to produce xylanase or lichenase when cultivated in the presence of apple or orange peels (Fig. 2).

Moreover, extracellular hemicellulolytic activities of the strain CCMM B940 were quantified using the dinitrosalicylic acid (DNS) spectrophotometric method for reducing sugars as described by Miller (1959). Protein concentration was determined by the Bradford protocol (Bradford 1976) with bovine serum albumin (BSA) as the standard. The amount of protein samples and the incubation time were chosen to provide assay conditions in which the measured activity was proportional to these two parameters. The enzymatic assays were all carried out at 55 °C to quantify the activities under conditions close to those of industrial processes using thermostable enzymes. Reducing sugars that were released after enzyme–substrate incubations were quantified in triplicates using glucose or xylose as a standard. One international unit (IU) was defined as the amount of enzyme, which produced 1 µmol of reducing sugars in 1 min. The xylanase and lichenase activities of the extracellular proteins from BSS cultures with 0.5% apple and orange peels are listed in Table 1. Strain CCMM B945 was again used as a control. No reducing sugars was detectable for strain CCMM B945 proteins incubated with xylan or lichenan. The lichenase activity of CCMM B940 was approximately two times higher than the xylanase activity in the cultures with natural peels. Both activities were higher in apple peel cultures than in orange peels, suggesting that the produced enzymes were either more numerous or more active in metabolizing carbohydrates in apple peels. This could be due to the nature of fibres present in the peel (sugar composition and types of glycosidic bonds) and/or to differential induction of glycoside hydrolases encoding gene expression. Strain CCMM B940 exhibited xylanase activities approximately twofold and over tenfold higher in apple peel cultures than thermophilic strains *B. licheniformis* 2D55 (Kazeem et al. 2017) and *Bacillus* sp. 275 (Gong et al. 2017), respectively.

**Table 1:** Lichenase and xylanase specific activities of extracellular proteins from *Bacillus* CCMM B940 and CCMM B945 cultivated in BSS medium supplemented with 0.5% apple and orange peels

Strains	Apple		Orange	
	CCMM B940	CCMM B945	CCMM B940	CCMM B945
Lichenase activity (IU/mg)	4.06 ± 1.1	nd	1.6 ± 0.2	nd
Xylanase activity (IU/mg)	2.39 ± 0.29	nd	0.98 ± 0.2	nd

Specific activities are given as µmol of glucose (incubation with lichenan) or xylose (incubation with xylans) equivalent produced per min and per mg of protein. Each value is the mean of three different assays (± SD)

*nd* not detectable

To further investigate the putative functions of the CCMM B940 strain and to achieve its reclassification within the genus *Bacillus*, we sequenced and examined the whole genome of this strain. Thus, genomic DNA was extracted with Wizard^®^ Genomic DNA Purification Kit (Promega, Charbonnières-les-Bains, France). The concentration and quality were determined using Nanodrop and Qubit spectrophotometers. Genomic DNA was sequenced at Eurofins Genomics (Cologne, Germany) using Illumina HiSeq technology. The library was prepared with 2 × 150-bp paired-end read length, including DNA fragmentation, adapter ligation, amplification, and size selection. All the steps were performed according to Eurofins Genomics protocols, producing 7,612,190 reads.

The raw reads were then trimmed using Trimmomatic v0.39 (Bolger et al. 2014) with the following parameters: SLIDINGWINDOW:4:20; ILLUMINACLIP: TruSeq3-PE.fa:2:30:20:2: LEADING:3, TRAILING:3 and MINLEN:60. We verified the presence/absence of phix sequences using bowtie2 (Langmead and Salzberg 2012) with the following parameters: –trim-to 80 –end-to-end –no-unal –no-sq –no-head –p 12. Processed reads were then assembled using the SPAdes de novo assembler v3.13.1 (Bankevich et al. 2012) testing assemblies with k-mer values: 21,33,55,77 using the parameter: –careful. Depending on the size of insert, scaffolds smaller than 350 bp were filtered out. Assembly quality was assessed using in-house script and QUAST v5.0.0 (Gurevich et al. 2013). We estimated genome completeness at 99.59% and checked that no contamination was detected using CheckM v1.1.3 (Parks et al. 2015).

**Fig. 3:**
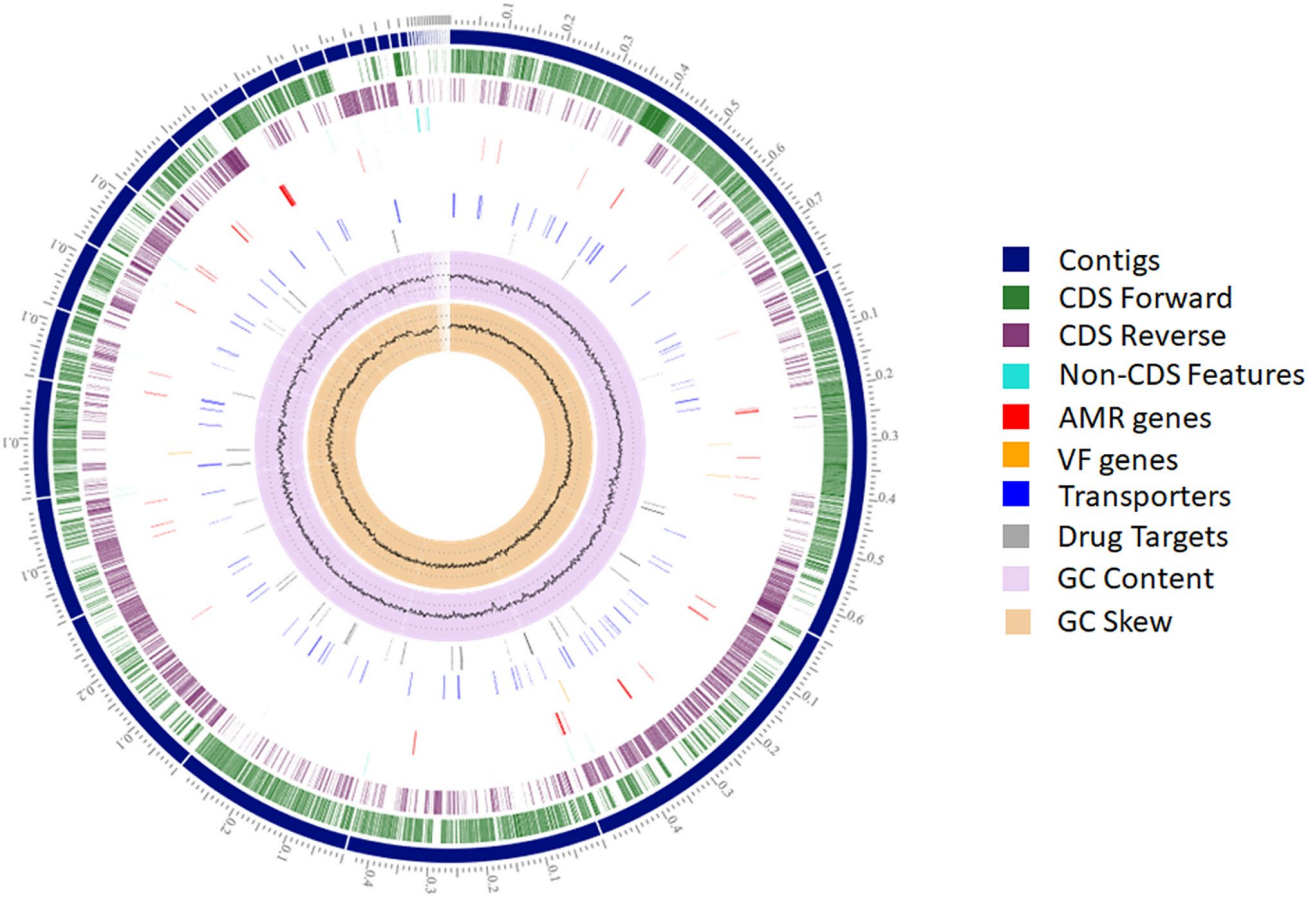
Circular representation of the *B. paralicheniformis* CCMM B940 chromosome using PATRIC based on 34 assembled scaffolds. From outer to inner ring—contigs (scale—x1Mbp), CDS on the forward strand, CDS on the reverse strand, RNA genes, homologous CDS to known antimicrobial resistance (AMR) genes, CDS with homology to known virulence factors (VF), GC content and GC skew

After eliminating low coverage and small contigs (< 350 bp), we obtained a total of 38 contigs and 34 scaffolds resulting in a genome of 4,315,004 bp (NCBI accession number JADRJL000000000) and an average G + C content of 45.87% which is close to other genomes from the *Bacillus subtilis* group (Rey et al. 2004; Du et al. 2019).The *N*50 of the draft genome is 440,121 bp, with an average of 126,912 bp and a notable four gap region (Fig. 3). Species identity was confirmed by comparing the genome reference sequences of *B. licheniformis* and *Bacillus paralicheniformis* available in the NCBI database (https://www.ncbi.nlm.nih.gov) using the BLAST service deployed on local servers with the genome obtained sequences and fastANI (Jain et al. 2018). The draft genome was most closely related to *B. paralicheniform*is Bac84 (accession number ASM299392v1) living in sea environment (Othoum et al. 2019) with an ANI value of 98.94%, and shared only 94.36% identity with *B. licheniformis* DSM 13^T^ (NC_006270.3). *B. aerius* CCMM B940 was, therefore, reclassified as *B. paralicheniformis* CCMM B940, placing it in the *B. subtilis* group; a group known for its wide array of uses in biotechnology mainly through species like *B. subtilis* and *B. licheniformis* (Fan et al. 2017). Taxonomic identity of the CCMM B940 strain was also confirmed using the GTDB-Tk tool for genome classification (Chaumeil et al. 2020) with 98.95% identity to the *B. paralicheniformis* KJ-16 genome (accession number ASM104248v2). The genome was then annotated using the default parameters of Prokka 1.14 (Seemann 2014) showing 4259 protein-coding genes, 51 tRNAs and 4 complete rRNAs.

**Fig. 4:**
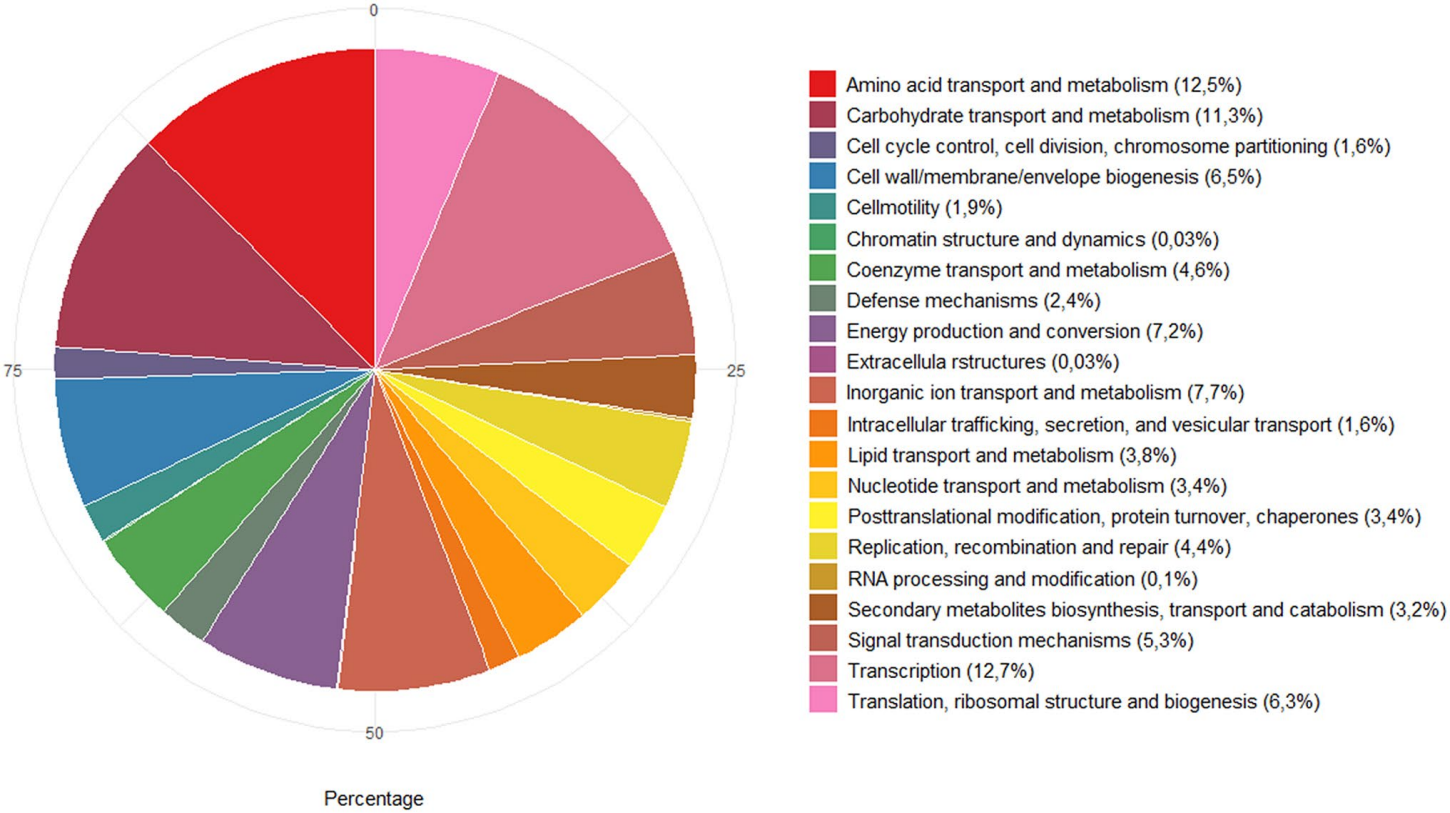
Distribution of biological functions of *B. paralicheniformis* CCMM B940 based on eggNOG analysis. Colors show different gene features categories and their proportions. Percentages of genes with known functions are given within parentheses

We further used eggNOG-mapper v2.0.1 (Huerta-Cepas et al. 2017) to assign proteins sequences into functional categories. More than 76% of the predicted proteins were attributed to functional subsystems. Proportions of genes with known functions (3047 CDS) were assigned to each category as presented in Fig. 4. Interestingly, a high proportion of CCMM B940 genes was assigned to the carbohydrate transport and metabolism (11.3%) as well as amino-acid transport and metabolism (12.5%) proteins. In a study conducted on *Bacillus velenzis*, *Bacillus safensis* and *Bacillus altitudinis* genomes, only 6.1% of the predicted proteins were assigned to carbohydrate transport and metabolism (Datta et al. 2020), while in *Bacillus amyloliquefaciens* and* Bacillus siamensis*, it represents a maximum of 8% in the pan and the core genomes (Chun et al. 2019). *B. paralicheniformis* has not been thoroughly studied for carbohydrate metabolism yet, but is phylogenetically very close to the *B. licheniformis* that has been widely used in the fermentation industry for production of enzymes, antibiotics and other chemicals (Madslien et al. 2012).

Hereafter, we focused on CCMM B940 glycoside hydrolases related to hemicellulose, cellulose and pectin degradation, comparing their protein sequences to the sequences of GH and pectate lyase (PL) of seven strains of *B. paralicheniformis* (A4-3, ATCC 9945a, BL-09, CBMAI 1303, FA6, MDJK30, ZAP17) available in the CAZY database (Lombard et al. 2014; database release 22 November 2020). Blast results was obtained using default parameters. Best hits with almost 90% amino-acid identity and 50% length alignment were selected and illustrated in Table 2 (the corresponding sequences are provided in Fig S2). Among 74 GH and PL sequences from CCMM B940 showed homology to other strains of *B. paralicheniformis*, 52 are dedicated to the degradation of carbohydrates in plant cell walls, representing 27 different CAZymes families according to the classification of B. Henrissat (Lombard et al. 2014). Thirty-two sequences of GH1, GH3, GH5, GH8, GH11, GH16, GH26, GH30, GH31, GH32, GH42, GH43, GH51, GH53, GH68, GH73 were linked to the degradation of hemicellulose, 4 sequences of GH5, GH9, GH12, GH48 for cellulose metabolism, 1 sequence (GH5) either for hemicellulose or cellulose degradation, and 11 sequences of GH (GH28, GH105) and PL (PL1, PL3, PL9, PL11, PL2) were attributed to pectin degradation (Table 2). Four sequences corresponded to enzymes (GH1, GH4) involved in glycolytic pathways. The remaining sequences were mainly related to the hydrolysis of starch and peptidoglycan (not shown).

**Table 2:** Annotated genes encoding lignocellulose-degrading enzymes in *B. paralicheniformis* CCMM B940

Enzymes	Accession number	Activity	CAZyme family	Reference genes		Identity (%)	Subject sequence length (bp)	Alignment length (bp)
				Accession number	Species			
Hemicellulose related	EOOONOAK_01749	Aryl-phospho-beta-d-glucosidase BglC	GH1	ARA87766.1	*Bacillus paralicheniformis* MDJK30	99.78	472	472
	EOOONOAK_03335	Aryl-phospho-beta-d-glucosidase BglH	GH1	AGN35231.1	*Bacillus paralicheniformis* ATCC 9945a	100	100	100
	EOOONOAK_03002	Aryl-phospho-beta-d-glucosidase BglC	GH1	AJO16530.1	*Bacillus paralicheniformis* BL-09	100	491	478
	EOOONOAK_01773	Aryl-phospho-beta-d-glucosidase BglH	GH1	QEO05069.1	*Bacillus paralicheniformis* A4-3	99.57	469	469
	EOOONOAK_03715	Endo-1,4-beta-xylanase A	GH11	QEO05734.1	*Bacillus paralicheniformis* A4-3	100	213	213
	EOOONOAK_02553	Beta-glucanase	GH16	ARA86799.1	*Bacillus paralicheniformis* MDJK30	99.59	243	243
	EOOONOAK_03322	Mannan endo-1,4-beta-mannosidase	GH26	QFY39913.1	*Bacillus paralicheniformis* FA6	98.89	360	360
	EOOONOAK_03205	Beta-hexosaminidase	GH3	QEO05378.1	*Bacillus paralicheniformis* A4-3	99.69	644	643
	EOOONOAK_02186	Glucuronoxylanase XynC	GH30	AJO20016.1	*Bacillus paralicheniformis* BL-09	100	420	420
	EOOONOAK_01418	Oligosaccharide 4-alpha-d-glucosyltransferase	GH31	QFY38704.1	*Bacillus paralicheniformis* FA6	99.75	802	802
	EOOONOAK_02225	Alpha-xylosidase	GH31	AJO19971.1	*Bacillus paralicheniformis* BL-09	99.61	769	769
	EOOONOAK_02112	Levanbiose-producing levanase	GH32	ARA87316.1	*Bacillus paralicheniformis* MDJK30	99.8	515	495
	EOOONOAK_01584	Sucrose-6-phosphate hydrolase	GH32	AJO20434.1	*Bacillus paralicheniformis* BL-09	99.37	478	478
	EOOONOAK_01729	Sucrose-6-phosphate hydrolase	GH32	AJO20595.1	*Bacillus paralicheniformis* BL-09	98.98	492	492
	EOOONOAK_00085	Levanase	GH32CBM66	QFY38018.1	*Bacillus paralicheniformis* FA6	100	677	677
	EOOONOAK_02224	Hypothetical protein	GH3CBM6	AJO19972.1	*Bacillus paralicheniformis* BL-09	100	980	980
	EOOONOAK_03904	Beta-galactosidase BglY	GH42	ARA84401.1	*Bacillus paralicheniformis* MDJK30	99.42	690	690
	EOOONOAK_04173	Beta-galactosidase YesZ	GH42	QFY39293.1	*Bacillus paralicheniformis* FA6	100	665	665
	EOOONOAK_01837	Beta-galactosidase GanA	GH42	QFY40673.1	*Bacillus paralicheniformis* FA6	99.56	684	684
	EOOONOAK_03858	Beta-xylosidase	GH43	ARA84797.1	*Bacillus paralicheniformis* MDJK30	99.62	532	532
	EOOONOAK_03859	Non-reducing end alpha-l-arabinofuranosidase	GH43	ARA84798.1	*Bacillus paralicheniformis* MDJK30	99.03	515	515
	EOOONOAK_03489	Extracellular endo-alpha-(1- > 5)-l-arabinanase 1	GH43	ARA85201.1	*Bacillus paralicheniformis* MDJK30	100	316	316
	EOOONOAK_00089	Beta-xylosidase	GH43	ARA86580.1	*Bacillus paralicheniformis* MDJK30	100	533	533
	EOOONOAK_01777	Extracellular endo-alpha-(1- > 5)-l-arabinanase 2	GH43	ARA88110.1	*Bacillus paralicheniformis* MDJK30	99.79	469	469
	EOOONOAK_02586	Extracellular endo-alpha-(1- > 5)-l-arabinanase 1	GH43	QFY37786.1	*Bacillus paralicheniformis* FA6	99.69	320	320
	EOOONOAK_02185	Arabinoxylan arabinofuranohydrolase	GH43CBM6	QFY37223.1	*Bacillus paralicheniformis* FA6	100	515	515
	EOOONOAK_01157	hypothetical protein	GH5	ARA85656.1	*Bacillus paralicheniformis* MDJK30	99.82	560	560
	EOOONOAK_02594	Intracellular exo-alpha-(1- > 5)-l-arabinofuranosidase	GH51	ARA86758.1	*Bacillus paralicheniformis* MDJK30	99.80	502	502
	EOOONOAK_01836	Arabinogalactan endo-beta-1,4-galactanase	GH53	AGN38579.1	*Bacillus paralicheniformis* ATCC 9945a	98.58	437	424
	EOOONOAK_02113	Levansucrase	GH68	ARA87315.1	*Bacillus paralicheniformis* MDJK30	100	481	481
	EOOONOAK_04229	Hypothetical protein	GH73	QEO06161.1	*Bacillus paralicheniformis* A4-3	98.95	570	570
	EOOONOAK_03432	Reducing-end xylose-releasing exo-oligoxylanase Rex8A xylanase probable	GH8	QFY39429.1	*Bacillus paralicheniformis* FA6	99.77	434	434
Cellulose-related	EOOONOAK_03622	Endoglucanase S	GH12	ARA86997.1	*Bacillus paralicheniformis* MDJK30	100	261	261
	EOOONOAK_01156	Exoglucanase-2	GH48	AJO18128.1	*Bacillus paralicheniformis* BL-09	100	717	704
	EOOONOAK_01385	Endoglucanase	GH5, CBM3	AJO18373.1	*Bacillus paralicheniformis* BL-09	99.81	518	518
	EOOONOAK_01155	Endoglucanase A	GH9CBM3	AJO18127.1	*Bacillus paralicheniformis* BL-09	100	653	653
Glucose metabolism	EOOONOAK_00372	6-Phospho-beta-glucosidase GmuD	GH1	AJO18923.1	*Bacillus paralicheniformis* BL-09	100	471	471
	EOOONOAK_01723	6-Phospho-beta-glucosidase GmuD	GH1	AJO20590.1	*Bacillus paralicheniformis* BL-09	99.16	478	478
	EOOONOAK_03054	Putative 6-phospho-beta-glucosidase	GH4	ARA84298.1 0	*Bacillus paralicheniformis* MDJK30	99.55	444	444
Hemicellulose or cellulose related	EOOONOAK_01655	Putative 6-phospho-beta-glucosidase	GH4	ARA87677.1	*Bacillus paralicheniformis* MDJK30	100	442	442
	EOOONOAK_01158	Hypothetical protein	GH5	QEO07050.1	*Bacillus paralicheniformis* A4-3	100	395	395
Pectin degradation	EOOONOAK_04163	Unsaturated rhamnogalacturonyl hydrolase YesR	GH105	AJO17709.1	*Bacillus paralicheniformis* BL-09	99.71	344	344
	EOOONOAK_02457	Unsaturated rhamnogalacturonyl hydrolase YteR	GH105	AJO19596.1	*Bacillus paralicheniformis* BL-09	99.73	373	373
	EOOONOAK_02256	Exo-poly-alpha-d-galacturonosidase	GH28	QFY37288.1	*Bacillus paralicheniformis* FA6	100	436	434
	EOOONOAK_02563	Hypothetical protein	PL1	QFY37765.1	*Bacillus paralicheniformis* FA6	100	494	494
	EOOONOAK_04252	Pectate lyase	PL1	AJO17744.1	*Bacillus paralicheniformis* BL-09	99.53	428	428
	EOOONOAK_01685	Pectate trisaccharide-lyase	PL1	QEO04986.1]	*Bacillus paralicheniformis* A4-3	99.71	341	341
	EOOONOAK_04170	Rhamnogalacturonan exolyase YesX	PL11	QEO06607.1	*Bacillus paralicheniformis* A4-3	100	628	628
	EOOONOAK_04168	Rhamnogalacturonan endolyase YesW	PL11	QEO08401.1	*Bacillus paralicheniformis* A4-3	100	622	622
	EOOONOAK_04174	Hypothetical protein	PL26	QEO06610.1	*Bacillus paralicheniformis* A4-3	99.07	886	863
	EOOONOAK_02080	Pectate lyase C	PL3	ARA87346.1	*Bacillus paralicheniformis* MDJK30	99.55	222	221
	EOOONOAK_01392	Hypothetical protein	PL9	QEO07258.1	*Bacillus paralicheniformis* A4-3	100	468	468

The accession numbers correspond to the CDS sequences annotated with Prokka as indicated in the manuscript. The corresponding sequences are provided in Figure S2

These predictions first confirmed the experimental observations in this study for the degradation of xylan, lichenan and CMC as the CCMM B940 genome carries glycoside hydrolases from GH5, GH9, GH11 and GH16 families of CAZYmes known to target these substrates. They also provided additional information on the carbohydrate metabolism of CCMM B940 such as pectinase activity as well as the presence of carbohydrate-binding modules (CBM3, CBM6, CBM66) associated with GHs helping them to bind to cellulose or hemicellulose polysaccharides to improve their efficiency. A few other studies (Wang et al. 2017; Chen et al. 2018) examined the genomic sequences of *B. paralicheniformis* and predicted their CAZymes, but without the experimental validation. To our knowledge, this is the first report that combines in silico genomic analysis and in vitro experiments to study the carbohydrate metabolism of *B. paralicheniformis.* These combined analyses reinforce the practical and potential use of CCMM B940 strain for industrial purposes. Indeed, *Bacillus* species are known to produce 60% of commercially available enzymes, mostly being homologous proteins that are naturally secreted in the growth medium (Westers et al. 2004). The strain CCMM B940 exhibits cellulolytic and hemicellulolytic activities at high temperature and produces extracellular thermostable enzymes.

Based on whole-genome sequencing, we demonstrated that the *B. aerius* strain CCMM B940 could be reclassified as *B. paralicheniformis*. Genomic analysis confirmed the fibrolytic potential of the strain CCMM B940 observed in vitro using purified and natural substrates and provided information on its ability to degrade pectins. This statement reinforces the strain's interest especially in agro-waste bioconversion. To our knowledge, this is the first report of lichenase and xylanase activities by a thermophilic strain of *B. paralicheniformis* isolated from a hot spring. The CCMM B940 strain is part of the large Coordinated Moroccan Collections of Microorganisms (CCMM), which comprise more than 1800 isolates of bacteria, yeasts and fungi. Many of these microorganisms have also been isolated from extremophile environments (Berrada et al. 2012; Aanniz et al. 2015) and they may harbor beneficial attributes such as production of heat-resistance enzymes as already observed in *B. amyloliquefaciens* (Beladjal et al. 2018) and *B. subtilis* (den Besten et al. 2017) strains. For this purpose, complete genome sequencing not only enables accurate identification and reclassification of CCMM isolates, i.e. the thermophilic strains of the *B. aerius* group, but also opens the door to the field of biotechnological applications such as production of second- and third-generation biofuels from raw or pre-treated biomass, medical and nutraceutical fields and food processing (Benedetti et al. 2019), in the favor of the fibrolytic potential (CAZymes) of these strains. Enzymatic activities of these strains should be further studied to determine the most suitable markets in biotechnology (agro-waste bioconversion, depollution, health, pharmaceuticals, etc.…).

### Accession numbers

The assembled genome and all relevant sequences were deposited in NCBI's GenBank on November 24, 2020 under the following accession numbers, BioProject PRJNA680612; BioSample SAMN16895636; Genome JADRJL000000000.

## Supplementary Information

Below is the link to the electronic supplementary material.

Fig. S1 Cultures of the CCMM B940 and CCMM B945 Bacillus strains in BSS medium supplemented with 0.5% orange or apple peels for 48h at 37°C. a. negative control without bacteria; b. growth (turbidity) is observed for cultures on both substrates (DOCX 1356 kb)

Fig.S2 CDS annotation of the genome of B.paralicheniformis CCMM B940 for lignocellulose-degrading enzymes (TXT 4743 kb)

## References

[CR1] Aanniz T, Ouadghiri M, Melloul M, Swings J, Elfahime E, Ibijbijen J, Ismaili M, Amar M (2015). Thermophilic bacteria in Moroccan hot springs, salt marshes and desert soils. Braz J Microbiol.

[CR2] Bankevich A (2012). SPAdes: a new genome assembly algorithm and its applications to single-cell sequencing. J Comput Biol.

[CR3] Beladjal L (2018). Life from the ashes: survival of dry bacterial spores after very high temperature exposure. Extremophiles.

[CR4] Benedetti M (2019). Green production and biotechnological applications of cellwall lytic enzymes. Appl Sci.

[CR5] Berrada I (2012). Diversity of culturable moderately halophilic and halotolerant bacteria in a marsh and two salterns a protected ecosystem of lower Loukkos (Morocco). Afr J Microbiol Res.

[CR6] Biely P, Singh S, Puchart V (2016). Towards enzymatic breakdown of complex plant xylan structures: state of the art. Biotechnol Adv.

[CR7] Bolger AM, Lohse M, Usadel B (2014). Trimmomatic: a flexible trimmer for illumina sequence data. Bioinformatics.

[CR8] Bouchaou L, Warner NR, Tagma T, Hssaisoune M, Vengosh A (2017). The origin of geothermal waters in morocco: multiple isotope tracers for delineating sources of water–rock interactions. Appl Geochem.

[CR9] Bradford MM (1976). A rapid and sensitive method for the quantitation of microgram quantities of protein utilizing the principle of protein-dye binding. Anal Biochem.

[CR10] Chaumeil PA, Mussig AJ, Hugenholtz P, Parks DH (2020). GTDB-Tk: a toolkit to classify genomes with the genome taxonomy database. Bioinformatics.

[CR11] Chen L (2018). Comparative genome analysis of *Bacillus velezensis* reveals a potential for degrading lignocellulosic biomass. 3 Biotech.

[CR12] Chun BH, Kim KH, Jeong SE, Jeon CO (2019). Genomic and metabolic features of the bacillus amyloliquefaciens group—B. Amyloliquefaciens, B. Velezensis, and B. Siamensis—revealed by pan-genome analysis. Food Microbiol.

[CR13] Cidu R, Bahaj S (2000). Geochemistry of thermal waters from Morocco. Geothermics.

[CR14] Datta S, Saha D, Chattopadhyay L, Majumdar B (2020). Genome comparison identifies different bacillus species in a bast fibre-retting bacterial consortium and provides insights into pectin degrading genes. Sci Rep.

[CR15] de la Torre I (2019). Utilisation/upgrading of orange peel waste from a biological biorefinery perspective. Appl Microbiol Biotechnol.

[CR16] den Besten HMW, Berendsen EM, Wells-Bennik MHJ, Straatsma H, Zwietering MH (2017). Two complementary approaches to quantify variability in heat resistance of spores of *Bacillus subtilis*. Int J Food Microbiol.

[CR17] Dhillon GS, Kaur S, Brar SK (2013). Perspective of apple processing 401 wastes as low-cost substrates for bioproduction of high value products: a review. Renewable 402 and sustainable energy reviews. Pergamon.

[CR18] Du Y (2019). Comparative genomic analysis of *Bacillus paralicheniformis* MDJK30 with its closely related species reveals an evolutionary relationship between *B. paralicheniformis* and *B. licheniformis*. BMC Genom.

[CR19] Dunlap CA (2015). The status of the species *Bacillus aerius*. Request for an opinion. Int J Syst Evol Microbiol.

[CR20] Fan B, Blom J, Klenk H-P, Borriss R (2017). *Bacillus amyloliquefaciens*, *Bacillus velezensis*, and *Bacillus siamensis* form an ‘Operational Group *B. amyloliquefaciens*’ within the *B. subtilis* species Complex. Front Microbiol.

[CR21] Gong G (2017). Complete genome sequence of *Bacillus* sp. 275, producing extracellular cellulolytic, xylanolytic and ligninolytic enzymes. J Biotechnol.

[CR22] Gurevich A, Saveliev V, Vyahhi N, Tesler G (2013). QUAST: quality assessment tool for genome assemblies. Bioinformatics.

[CR23] Houfani AA (2020). Insights from enzymatic degradation of cellulose and hemicellulose to fermentable sugars: a review. Biomass Bioenergy.

[CR24] Huerta-Cepas J (2017). Fast genome-wide functional annotation through orthology assignment by EggNOG-mapper. Mol Biol Evol.

[CR25] Jain C (2018). High-throughput ANI analysis of 90K prokaryotic genomes reveals clear species boundaries. Nat Commun.

[CR26] Joglekar SN, Pathak PD, Mandavgane SA, Kulkarni BD (2019). Process of fruit peel waste biorefinery: a case study of citrus waste biorefinery, its environmental impacts and recommendations. Environ Sci Pollut Res.

[CR27] Kazeem MO, Shah UKM, Baharuddin AS, AbdulRahman NA (2017). Prospecting agro-waste cocktail: supplementation for cellulase production by a newly isolated thermophilic *B. licheniformis* 2D55. Appl Biochem Biotechnol.

[CR28] Knapik K, Becerra M, González-Siso MI (2019). Microbial diversity analysis and screening for novel xylanase enzymes from the sediment of the lobios hot spring in Spain. Sci Rep.

[CR29] Langmead B, Salzberg SL (2012). Fast gapped-read alignment with bowtie 2. Nat Methods.

[CR30] Lombard V (2014). The carbohydrate-active enzymes database (CAZy) in 2013. Nucleic Acids Res.

[CR31] Madslien EH, Olsen JS, Granum PE, Blatny JM (2012). Genotyping of *B. licheniformis* based on a novel multi-locus sequence typing (MLST) scheme. BMC Microbiol.

[CR32] Miller GL (1959). Determination of reducing sugar by DNS method. Anal Chem.

[CR33] Othoum G (2019). Comparative genomics study reveals Red Sea Bacillus with characteristics associated with potential microbial cell factories (MCFs). Sci Rep.

[CR34] Parab P, Khandeparker R, Amberkar U, Khodse V (2017). Enzymatic saccharification of seaweeds into fermentable sugars by xylanase from marine *Bacillus* sp. strain BT21. 3 Biotech.

[CR35] Parks DH (2015). CheckM: assessing the quality of microbial genomes recovered from isolates, single cells, and metagenomes. Genome Res.

[CR36] Patrascu O (2017). A fibrolytic potential in the human ileum mucosal microbiota revealed by functional metagenomic. Sci Rep.

[CR37] Rey MW (2004). complete genome sequence of the industrial bacterium *Bacillus licheniformis* and comparisons with closely related *Bacillus* species. Genome Biol.

[CR38] Rivas B (2008). Submerged citric acid fermentation on orange peel autohydrolysate. J Agric Food Chem.

[CR39] Seemann T (2014). Prokka: rapid prokaryotic genome annotation. Bioinformatics.

[CR40] Thapa S (2020). Microbial cellulolytic enzymes: diversity and biotechnology with reference to lignocellulosic biomass degradation. Rev Environ Sci Biotechnol.

[CR41] Wang Y (2017). Complete genome sequence of *Bacillus paralicheniformis* MDJK30, a plant growth-promoting rhizobacterium with antifungal activity. Genome Announc.

[CR42] Westers L, Westers H, Quax WJ (2004). *Bacillus subtilis* as cell factory for pharmaceutical proteins: a biotechnological approach to optimize the host organism. Biochimica Et Biophysica Acta (molecular Cell Research).

[CR43] Zeng Y, Himmel ME, Ding SY (2017). Visualizing chemical functionality in plant cell walls mike himmel. Biotechnol Biofuels.

[CR44] Zhang Z-Y (2018). Complete genome sequence of *Bacillus velezensis* ZY-11 reveals the genetic basis for its hemicellulosic/cellulosic substrate-inducible xylanase and cellulase activities. 3 Biotech.

