# Supplementary material for: Hemicellulosic biomass conversion by Moroccan hot spring Bacillus paralicheniformis CCMM B940 evidenced by glycoside hydrolase activities and whole genome sequencing
Source: 3 Biotech. 2021 Jul 22;11(8):379. doi: 10.1007/s13205-021-02919-0 (PMC8298745; doi:10.1007/s13205-021-02919-0)
Supplement: Supplementary file 2 — Fig.S2 CDS annotation of the genome of B.paralicheniformis CCMM B940 for lignocellulose-degrading enzymes (TXT 4743 kb) [file 13205_2021_2919_MOESM2_ESM.docx]

Fig.S1
